# Design and Manufacture of Multifunctional 3-D Smart Skins with Embedded Sensor Networks for Robotic Applications

**DOI:** 10.3390/s24113441

**Published:** 2024-05-27

**Authors:** Elliot Ransom, Xiyuan Chen, William Mangram, Amir Nasrollahi, Tanay Topac, Fu-Kuo Chang

**Affiliations:** 1 Department of Aeronautics and Astronautics, Stanford University, Stanford, CA 94305, USA; willmngrm@gmail.com (W.M.); amirnasr@stanford.edu (A.N.); tanaytopac@gmail.com (T.T.); 2 Department of Mechanical Engineering, Stanford University, Stanford, CA 94305, USA; xiyuan@stanford.edu

**Keywords:** sensor, network, smart structure, PZT, deployment, design, optimization

## Abstract

An investigation was performed to develop a process to design and manufacture a 3-D smart skin with an embedded network of distributed sensors for non-developable (or doubly curved) surfaces. A smart skin is the sensing component of a smart structure, allowing such structures to gather data from their surrounding environments to make control and maintenance decisions. Such smart skins are desired across a wide variety of domains, particularly for those devices where their surfaces require high sensitivity to external loads or environmental changes such as human-assisting robots, medical devices, wearable health components, etc. However, the fabrication and deployment of a network of distributed sensors on non-developable surfaces faces steep challenges. These challenges include the conformal coverage of a target object without causing prohibitive stresses in the sensor interconnects and ensuring positional accuracy in the skin sensor deployment positions, as well as packaging challenges resulting from the thin, flexible form factor of the skin. In this study, novel and streamlined processes for making such 3-D smart skins were developed from the initial sensor network design to the final integrated skin assembly. Specifically, the process involved the design of the network itself (for which a physical simulation-based optimization was developed), the deployment of the network to a targeted 3D surface (for which a specialized tool was designed and implemented), and the assembly of the final skin (for which a novel process based on dip coating was developed and implemented.)

## 1. Introduction

### 1.1. Background

The smart geometry created during this study is an example of a smart structure, one functionalized with a smart skin. A smart skin is a component that can be used as the sensing “organ” of a smart structure; smart structures use integrated sensors, actuators, and control elements to respond adaptively to their environments [1]. These structures take on various scales, forms, and applications, and are used in a variety of disciplines from structural health monitoring to medical health monitoring [2,3,4,5,6,7,8,9,10,11,12,13,14].

The smart skin is distinguished by its form factor, which is similar to that of the human skin organ in its robustness, ability to conformally cover complex contours and geometries, and its multifunctional sensing ability, allowing the skin to provide important environmental data. Of special interest relating to the human skin are hair follicles, Meissner corpuscles, Pacinian corpuscles, Merkel discs, Ruffini corpuscles, and thermoreceptors; between them, these different sensors can sense touch, slip, vibration, texture, stretch, and temperature [15].

The aspiration of the smart geometry created in this study is to provide a platform for multifunctional smart structures that can be applied to biomimetic structures like a mimetic human finger. Ultimately, it is desired that the smart skin designed as part of this study be applicable to a wide variety of biomimetic geometries, particularly ones germane to humanoid robots.

### 1.2. Motivation

In general, smart skin platforms are useful in applications where the geometry of the object to be functionalized is non-developable; that is, it is doubly curved. These applications include the following: the control of flexible, compliant geometries such as soft manipulators and morphing wings [16,17], medical devices such as organ monitoring “sleeves” and prostheses [12,18], and wearable devices for detecting external environmental hazards and data gathering [19,20,21].

Above all, smart skin capabilities provide vital environmental data in a package compatible with humanoid geometries, allowing robots the ability to understand and interact with their surroundings in a variety of ways [22,23,24,25,26,27,28]. For instance, a smart skin with pressure sensors could allow for the localization and quantification of pressure forces in contact with the skin, permitting safe interactions with humans in the robot’s operating environment, such as a human being able to brush a robot’s arm aside. Similarly, smart skins have the potential to fuse multiple sensor modalities to allow for the identification and discrimination of different textures, the identification of the compliance of an object to control grasping force, the detection of slip against the robot’s skin to avoid the dropping of held objects, and temperature recognition, as well as the avoidance of hot obstacles. These capabilities are especially important in robots designed to interact with humans in an uncontrolled environment, such as nursing robots.

### 1.3. Problem Statement

While the smart skin platform is a compelling one, several technical hurdles exist that must be addressed in its development: requirements on the positional accuracy of the deployed sensors, as well as their functionality, packaging, and integration. The smart skin must conformally cover the target object; in many cases, this object may be doubly curved. Before this work, no non-developable surface had been functionalized using the Stretchable Sensor Network (SSN) developed at the Structures and Composites Lab (SACL).

**Figure 1:**
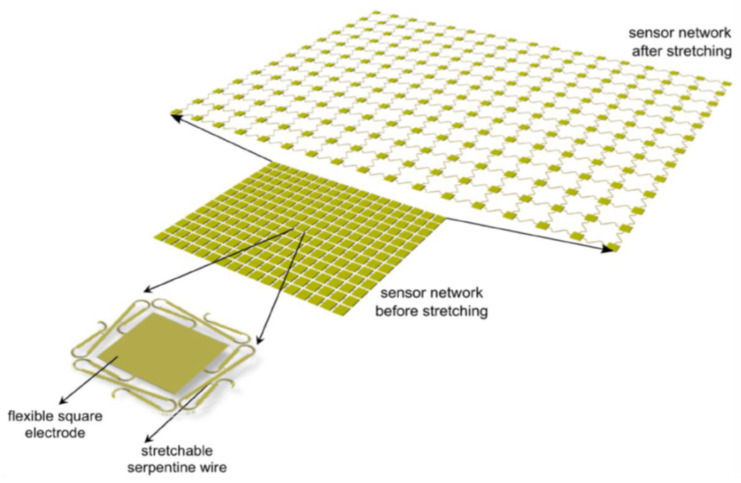
The SSN concept is shown.

The SSN used at the Structures and Composites Lab (pictured in Figure 1) provides a compelling platform for distributed sensing and has been applied in multiple contexts to provide distributed sensing on developable surfaces such as airfoils and satellite panels [29]. The integration of this SSN into a non-developable object poses major obstacles, which we examine in the following paragraphs.

An SSN deployed in 3-D must be wrapped around a target object, not merely projected straight down onto a plane. As such, a method must be devised for designing an SSN in 2-D such that the sensor nodes occupy targeted locations in 3-D after deployment. The hardware for carrying out the deployment method itself must also be devised, one that allows the network to be physically wrapped around the object.

## 2. Related Work

In the following subsection, some state-of-the-art approaches to solving the doubly curved deployment problem are examined.

One simple approach to the problem of covering a doubly curved surface with a substrate involves cutting that substrate into small patches that conform locally to the surface. Indeed, objects such as soccer balls and baseballs are fabricated from tessellated patches, and this principle has been used to cover arbitrary geometries [30]. Perhaps the most prominent work of this type is the ROBOSKIN of the iCub robot developed by The Italian Institute of Technology in 2012, in which triangular capacitive sensing modules on flexible printed circuit boards (PCBs) were tessellated to form patches embedded in a robotic skin [31].

The primary issue with this approach is that it is fundamentally limited by the radius of the curvature of the object to be covered. This is because the patches must be small enough to locally conform to the object; if an object has a very low radius of curvature, as in the case of a finger, the patches must be prohibitively small in order to cover the curved surface of the object, or the individual fingertips must be functionalized separately as in the case of iCub [30]. Because of this, work of this type typically employs patches to cover areas with radii of curvature that are large relative to the areal footprint of the functionalized sensors.

Another approach to doubly curved surface coverage is to use a flexible substrate capable of withstanding enough strain to wrap the object. Substrates such as PDMS and silicone are examples of materials that have been used in such approaches [32,33,34].

Several issues present themselves here as well. The key problem with this approach is that it involves first embedding the skin electronics in the flat substrate and then attempting to cover the target geometry in the resulting flat skin. While this allows the conformal coverage of relatively gently curved surfaces, this approach is not appropriate for geometries where the flat skin is overly strained during the deployment process. Too much strain in these cases can result in the substrate itself failing, or the embedded electronics failing. Additionally, flexible substrates can wrinkle as well. As such, this approach is not appropriate for geometries with small radii of curvature or sheer vertical geometries (imagine protruding a finger through a silicone skin and visualizing the resulting strain). Like in patch-based approaches, flexible substrates are typically used in situations where the target geometry is nearly developable, and sometimes, these substrates are even split into patches as well.

Aside from conforming to a doubly curved geometry, the smart skin must be packaged carefully. That is, all of the sensors and interconnects must be embedded inside the skin itself. At the same time, the skin must be integrable with other systems such as data acquisition and control systems, requiring sensors to be wired out of the skin or for data to be wirelessly transmitted, and even in the wireless transmission case, on-board power must be generated in the skin or wired into the skin.

In addition to the above obstacles, a smart skin must contain sensors in prescribed locations on the target object. In previous work where the skin approach was not present, it was relatively simple to install individual sensors in precise locations and then wire them directly to other systems. While appropriate when few sensors are installed, this approach is not very scalable and can inhibit the operation of the target device through the inclusion of complex wiring apparatuses.

When a skin approach is used, by far, the most common approach is to fabricate sensor networks in a regular pattern to ensure that the entire skin area is functionalized. However, there are example use cases in which a variable sensor density may be desired. An example would be a hand-like manipulator, which would require an increased sensor density in the fingertips relative to other parts of the manipulator, such as the back of the “hand” of the manipulator.

## 3. Proposed Method

The definition of a smart skin must be revisited: a smart skin is a sensing component comprising a network of sensors distributed at specific locations inside a soft polymer skin. This means that several problems must be tackled in sequence: the sensor network must be designed such that individual sensors are distributed at specific locations; the sensor network must be deployed at the target locations on a doubly curved surface; the sensor network must be embedded within a soft, compliant skin, again on a doubly curved surface; and the smart skin must be functionalized by a connection to a data acquisition unit.

The target part should have the following two attributes to make it an appropriate demonstrator for the technology. First, it should contain a doubly curved surface with a sufficiently small radius of curvature to demonstrate the conformal coverage of a doubly curved surface. Second, it should contain a sheer vertical geometry that would be prohibitive to protrude through a flexible substrate. The target part can be conceptualized as a robotic manipulator, control structure, body part, or doubly curved surface to be monitored structurally; given this target part, how can a smart skin be created that sensors are located at prescribed locations on the surface of the object?

**Figure 2:**
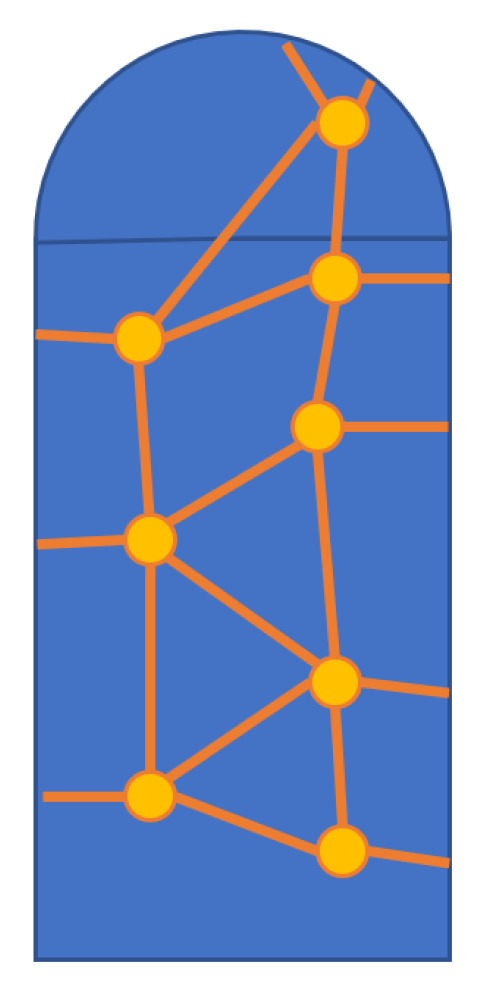
A simple target part with a sensor network is shown.

**Figure 3:**
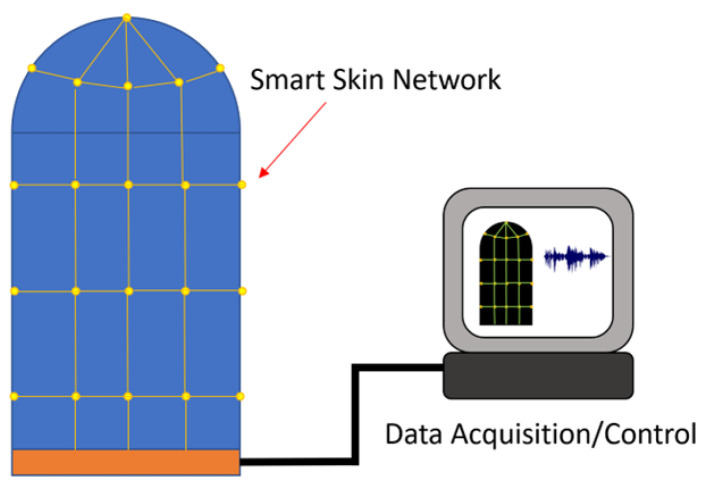
A functionalized smart skin is connected to a data acquisition unit.

This work pursued the above problem as applied to a specific part: a cylinder with a hemispherical end cap, pictured in Figure 2. This satisfies the doubly curved and sheer vertical geometry requirements outlined above, and the smart skin designed during this work conformed to this design part. The functionalized skin must also be wired to a data acquisition unit as pictured in Figure 3.

## 4. Method of Approach

### 4.1. Overview

The smart skin proposed in this study comprises three key features: a network of distributed sensors deployed at specific locations inside the skin, a soft polymer skin in a 3-D double curvature configuration to host the sensors and protect the sensor network, and an integrated system that can output temperature, strains, and dynamic impact on the skin from embedded sensors.

**Figure 4:**
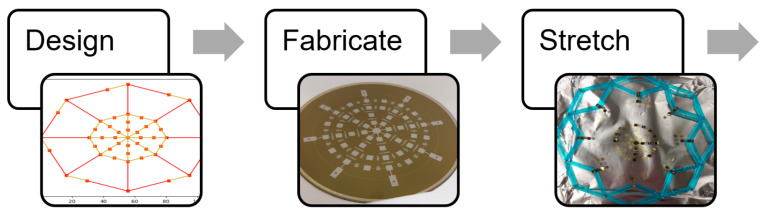
The process for creating a smart skin: design through network stretch.

**Figure 5:**
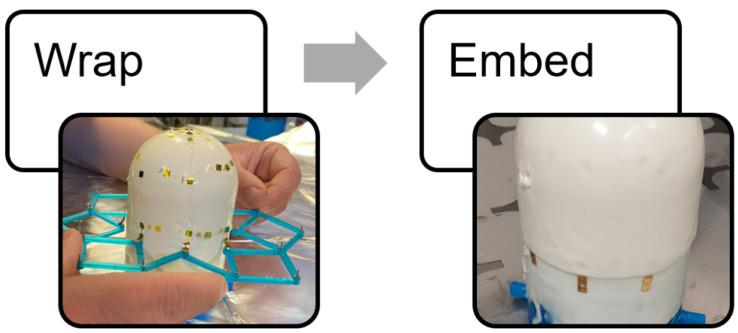
The process for creating a smart skin: wrap and embed steps.

To achieve the outcomes above, a five step process is proposed. The network was first designed and then fabricated on a 100 mm wafer. This network was released from the wafer and then stretched to its design size using a specialized tool. This same tool was used to wrap the network around a target object. Finally, the wrapped network was embedded in a skin and connected to data acquisition devices. The process is summarized in Figure 4 and Figure 5.

In this section, the steps for the successful development of the smart skin are discussed in sequence.

### 4.2. Theory and Design

The first method developed in this study concerns the design of a network that is capable of satisfying the positional requirements on its sensors. Given a symmetric network with interconnect stiffness values $k_{1},k_{2},\ldots ,k_{n}$, a method must be developed for finding the appropriate $k_{i}$ that allows the network to be deployed on the target object while satisfying these positional requirements. This requires the completion of the following tasks:

- The determination of target sensor locations.
- The back-design of wire interconnects to satisfy these target locations.
- The manufacture of the sensor network in the SNF.

**Figure 6:**
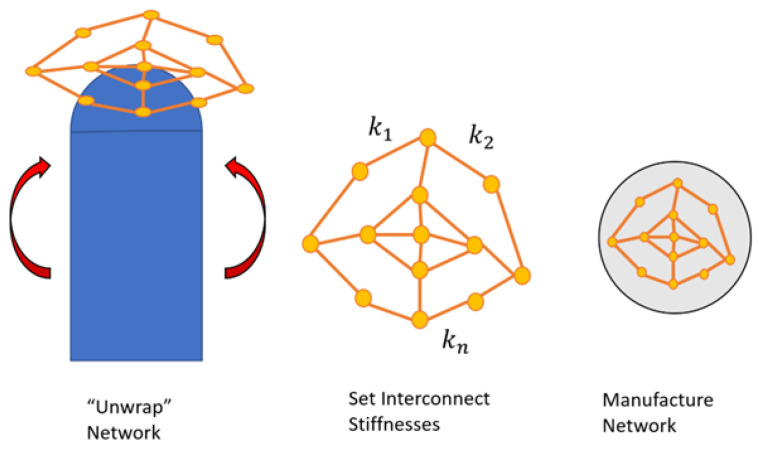
The “unwrapped” network is designed and fabricated.

The SSN must be designed such that, when it is deployed, the sensors are in the required locations. Sensor locations can be adjusted by changing the stiffness of the wires that connect these sensors together. This can be cast as an optimization problem where the wire stiffness values that minimize the error in the resulting sensor locations are desired. Figure 6 shows the approach of designing the 2-D footprint based on the 3-D target locations.

To solve this optimization problem, two things are required. First, a physical model that determines where the sensors will end up after an SSN with given wire stiffness values is deployed. Second, an optimization algorithm that takes the results of the physical simulations and converges to the optimal design. In this work, two tools were developed: a simulation method and a particle swarm optimization method.

Specifically, the simulation method used in this work is based on the principle of minimum energy. In this simulation, each wire (or “interconnect”) was modeled as a linear spring, each with its own displacement and stiffness (the $k_{i}$) and, by extension, its own contribution to the total potential energy of the entire SSN system. This is itself an optimization problem, as the positions of each node in the SSN system must be selected to minimize the total potential energy of the system at each step of the simulation. During the simulation, the nodes at the edge of the network are moved very slightly, and then the remaining node positions are determined through the principle of minimum energy. This process is repeated until the network has been fully deployed.

The optimization algorithm used in this work is based on particle swarm optimization. In this scheme, several candidate solutions are simulated, each one producing a corresponding particle in the search space. Over several iterations, the velocity and positions of these particles in the search space are manipulated based on the best-known locations in the search space, ideally causing the particles to converge. The advantage of this approach is that it does not require the gradient of the problem to be calculated; this is essential, as the problem does not have a closed-form solution, and calculating the gradient differentially is prohibitively computationally expensive.

In the particle swarm optimization technique used in this work, the “particles” to be optimized were sets of interconnect stiffness values (“sets”). A number of random sets were simulated using the aforementioned physical simulation, and the resulting sensor locations of the simulation were compared to the desired locations, serving as a loss function. The tendency of the particle swarm is to navigate the search-space and converge on the optimal set.

The resulting optimal network was fabricated at the Stanford Nanofabrication Facility (SNF).

#### 4.2.1. Physics-Based Simulation

In this section, the method for simulating the deployment of a sensor network with a given set of stiffness values is elaborated on.

At its heart, the SSN comprises a set of nodes connected to one another by serpentine interconnects, and this fact governs the physical simulation. Namely, the SSN can be represented as a network of nodes connected by springs. In this conceptualization, each interconnect is assigned a stiffness value and is assumed to behave as a linear spring during simulation. In fact, this assumption is valid for much of an SSN interconnect’s stretching process, during which it is assumed that all nodes on the perimeter of the network are positionally constrained to move along predetermined paths.

Finally, the behavior of a given network node as it comes into contact with the design object must be simulated. The surface characteristics of the design object are described in Section 6, but for now, it is assumed that a node that contacts the design object will remain in contact with the design object at the location of its contact. That is, nodes do not move after realizing contact with the object or “slip” against the object in any way.

In order for it to be performed multiple times during the optimization process, the simulation was designed to be as lightweight as possible. First, a “network object” consisting of a network of springs and nodes is initialized. Interconnect stiffnesses are prescribed by an initial guess or a previous optimization loop. Then, the simulation proceeds as follows:

1. The edge nodes are moved some small distances along their predetermined paths, and their positions are updated in the simulation.
2. The positions of the interior nodes are calculated. This is accomplished by performing an L-BFGS-B optimization.
3. Nodes are checked for collision with the target geometry. If a node is found to be in contact with the target geometry, it is fixed at the point of contact for the remainder of the simulation.
4. Steps 1–3 are repeated until the edge nodes have reached the ends of their predetermined paths.

At the conclusion of the simulation, the squared positional error between the targeted and simulated sensor locations is used as a loss function for the purposes of optimization.

#### 4.2.2. Optimization

A number of optimization algorithms were considered for use in this investigation, starting with gradient descent algorithms. However, during experimentation, it became clear that the bottleneck in the optimization was the runtime of the simulation; the simulation needs to run multiple times for the optimization routine to proceed, and in gradient descent, the simulation must run a prohibitive number of times. To combat, this, particle swarm optimization (PSO) was employed, which reduced the number of required simulations from tens of thousands to merely hundreds. The particle swarm optimization technique features robustness and is applicable to a variety of engineering problems [35,36].

Particle swarm optimization (PSO) derives its name from its use of “particles” in the parameter space that “swarm” around an optimum. In this case, the parameter space is the set of possible stiffness values that could be selected, and the optimum is the set of stiffness values that minimizes the loss function. The technique, as implemented in this investigation, follows the procedure outlined as follows:

1. A population of “guesses” in the parameter space is generated. In these initial guesses, the values of the spring stiffness are generated randomly. These ”guesses” form the set of particles, and their parameter values are their “positions” in the parameter space.
2. Each particle is evaluated for fitness by running the aforementioned physical simulation corresponding to its parameters. The simulation is scored based on the loss function.
3. The resulting particles are sorted by their fitness score after they have all been evaluated.
4. The positions of the fittest particles in the simulation history are averaged to generate a “center of gravity”; that is, a point in the parameter space that attracts the particles.
5. The particles are given “velocities” in the parameter space with terms corresponding to a decaying “inertial” term proportional to the current velocity of the particle and a term proportional to the distance from the center of gravity of the particle.
6. The positions of the particles are updated based on the given velocities.
7. This loop from the evaluation to position update repeats for several iterations until the particles swarm around a suitable optimum.

In addition to runtime, the exploration of the parameter space is an important consideration when tuning hyperparameters for the PSO algorithm. Namely, those of interest are as follows:

- The coefficients of the velocity terms;
- The size of the weighted average used to determine the center of gravity;
- The numbers of iterations and particles.

In general, the velocity assigned to a given particle during each iteration is given as

(1) $$v_{k+1}=c_{r}\left(1-\frac{k}{n}\right)+c_{c}\left(x_{c}-x_{k}\right)+R$$

where the variables are defined as follows:

- *k* is the iteration number of the routine;
- *v* is the velocity of the particle;
- *x* is the position of the particle;
- $c_{r}$ is the coefficient corresponding to the inertial term;
- $c_{c}$ is the coefficient corresponding to the center-of-gravity term;
- *n* is the total number of iterations;
- *R* is a random adjustment to the velocity value.

Note that the inertial term of the velocity decays, causing the particles to tend to converge as the simulation progresses.

In examining this equation, some behaviors that may occur depending on parameter selection can be described. For instance, if $c_{r}$ is set to be relatively very large compared to $c_{c}$, particles will continue along their current trajectories without being affected by the center of gravity, exploring more space but tending not to converge to a solution. If the reverse is set, then particles will be attracted toward the center of gravity strongly, resulting in relatively little exploration and faster convergence. After sweeping both parameters though example configurations of the PSO code, coefficients of 1.2 and 1.2 were used for the velocity coefficient hyperparameters.

Another hyperparameter that affects the exploration of the parameter space is the size of the weighted average used to determine the center of gravity of the PSO swarm. This center of gravity is the average position in the parameter space of the most fit particles discovered during the optimization, and the number of particles considered in this average were also tuned through experimentation.

Considering edge cases can illuminate the necessity of tuning here. In the case where only one particle is considered, less exploration is conducted; this results from the fact that only the fittest particle is considered, ignoring all other possibly fit solutions. Conversely, considering every particle in the simulation would be too exploratory, resulting in convergence to the mean position of all particles in parameter space rather than a controlled convergence to an especially fit solution or optimum.

After tuning over multiple simulations, a value of 5 was determined for the size of the weighted average in this simulation.

### 4.3. Design Results

**Figure 7:**
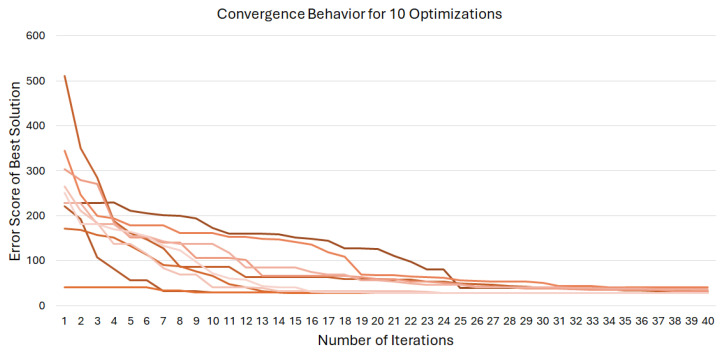
The convergence behaviors for 10 optimization runs are shown.

Because the particle swarm algorithm is initialized with random particles, it is important that it be run multiple times so as to ensure the solution reached is not simply a local minimum. The PSO algorithm was run 10 times; the convergence behaviors for the 10 PSO runs are shown in Figure 7.

In this figure, it is shown that the 10 simulations have slightly varying convergence behaviors but converge to optimum values within 25 iterations of the start of the PSO routine. In addition, the error function value of the optimum approaches, but does not reach, zero. This implies that even at the optimal solution, there is some error between the designed and simulated sensor node positions.

**Figure 8:**
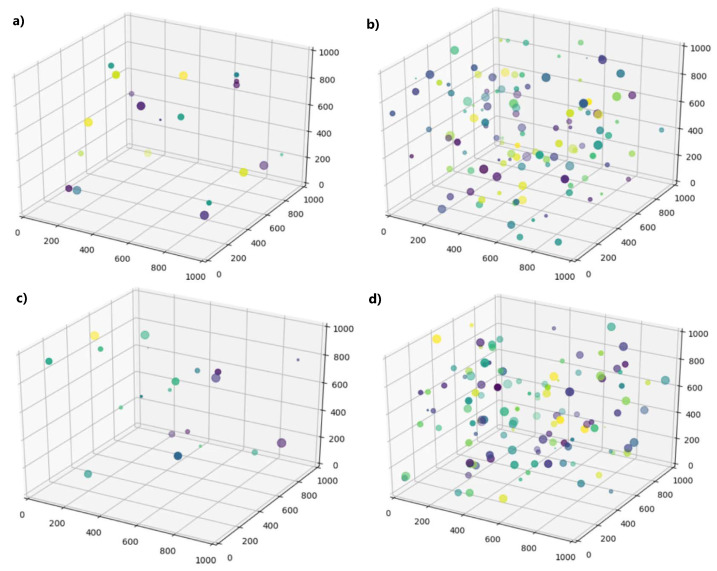
Time lapse of a 20 population simulation with corresponding error scores at (**a**) the initial generation, (**b**) 5 iterations, (**c**) 10 iterations, and (**d**) 20 iterations. Particle color and size denote error scores in the 4th and 5th dimensions, and are included for qualitative comparison.

Because the optimization routine optimizes a problem in five parameters, the particle swarm optimization process can be plotted visually using a scatter plot by assigning three parameters to positional values in 3-D, one parameter to dot size, and one parameter to dot color. One representative simulation is shown in Figure 8.

It can be seen that these simulations converge to similar optima, except for in the parameter corresponding to the color dimension. It was found that changing this parameter had no effect on the final configuration of the network; this is a consequence of statics and the symmetric design of the network.

**Figure 9:**
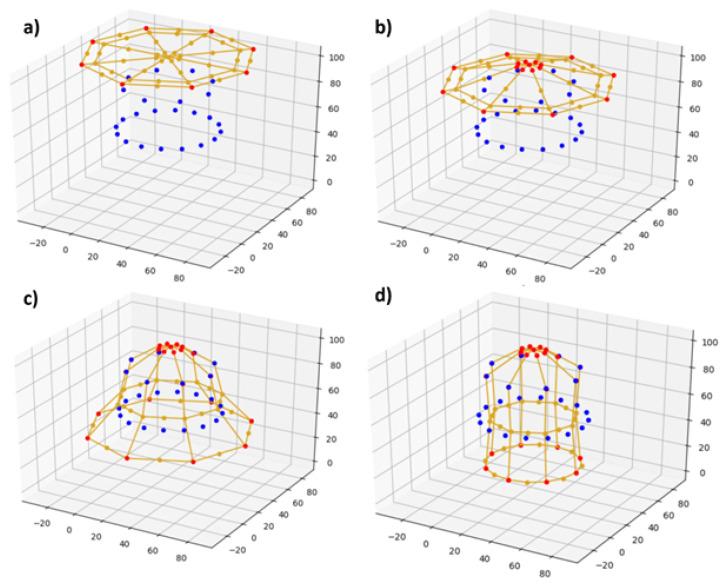
Time lapse of the simulation of the optimal network at (**a**) initial deployment, (**b**) first contact with the hemisphere, (**c**) the complete extension of the network, (**d**) the final configuration of the deployed network. Red points indicate fixed points moved by the simulation, blue points represent target locations, and yellow points indicate sensor locations that are not fixed.

To further illustrate the PSO results, an optimal network can be simulated and visualized as it is deployed over the target object. Figure 9 provides such a visualization.

It can be seen that the simulated optimal network very nearly overlaps the target locations for the network, but there is one minor discrepancy. Specifically, nodes in the third ring of the network appear to be offset from their target locations radially. To explain this, the collision physics of the simulation must be examined. To ensure that the simulation was lightweight, the interconnects in this simulation have no collision physics, allowing them to clip through the collision geometry. Likewise, to simplify the collision geometry, sidewalls were excluded from this geometry.

In knowing these facts, the discrepancy can be explained. During the simulated deployment, the second ring of sensors comes into contact with the hemisphere, and the relevant nodes collide with the object and are fixed in place. The deployment simulation then continues until it is completed, with the nodes in the fourth ring coming to rest at the surface of the target object. However, because of the lack of collision physics on the sidewall of the target object, the nodes in the third ring equilibrate somewhere between those in the second and fourth rings along a path that clips through the geometry. In spite of this discrepancy, the error in this configuration is so small as to be negligible, and the optimal design determined by the 2-D-to-3-D design tool was selected for manufacture.

At this stage of the design process, the relative stiffness values of each interconnect in the optimal network are known. This information must be used to design interconnects with appropriate geometries to reproduce these relative stiffness values.

**Figure 10:**
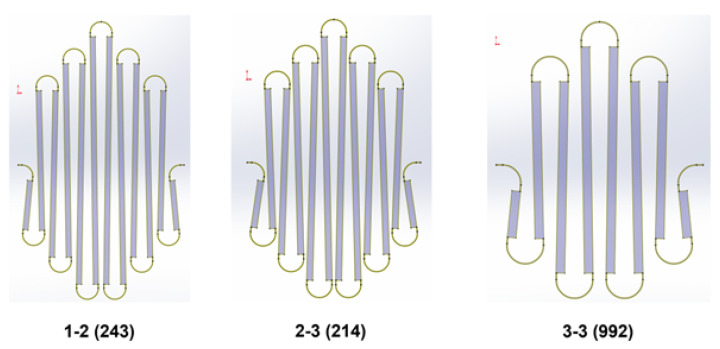
Three interconnect geometries achieving three different stiffness values were generated.

Through FEM simulation, SACL has previously developed a library of interconnects of varying stiffness values that have been shown to be effective in 2-D applications, as demonstrated in [10,11]. Interconnects from this library were selected to form the interconnect geometries used in the design of the optimal network. Figure 10 shows some example network geometries.

**Figure 11:**
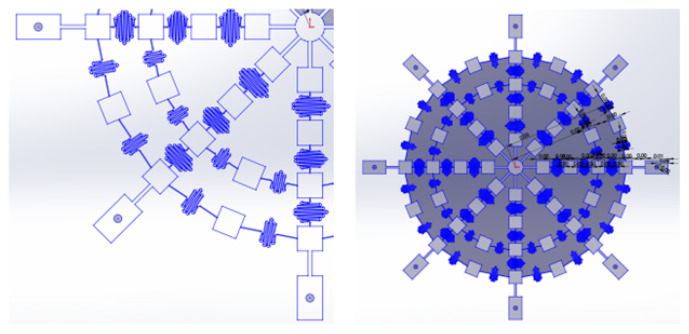
The completed network design is shown.

With these geometries in hand, the geometry of the entire network can be determined, pictured in Figure 11.

## 5. Experimental Procedure

After the sensor network is fabricated, it must then be deployed and embedded within a compliant skin. In this section, the fabrication of and experimentation on the smart skin is detailed.

### 5.1. SSN Microfabrication

The SSNs fabricated in this study contained footprints for resistive temperature dectors (RTDs) and lead ziconate titanate (PZT) sensors; while the RTD sensors were manufactured in a cleanroom at the Stanford Nanofabrication Facility, the PZTs were attached to patterned electrodes after the cleanroom process was complete.

The process used to manufacture the SSNs was detailed in previous work by SACL [10,11], and is summarized below. All steps were performed on a 100 mm silicon wafer:

1. A silicon dioxide etch stop layer was created using low-pressure chemical vapor deposition.
2. A germanium sacrificial layer was deposited via e-beam evaporation.
3. A polyimide layer, which serves as the bottom encapsulation layer of the finished SSN, was spin-coated on the wafer.
4. Titanium (for the RTD-sensing elements) and gold (for the signal layer and PZT electrodes) were deposited over a patterned photoresist mask.
5. The top encapsulating layer of polyimide was spin-coated onto the wafer.
6. An aluminum etch mask was patterned, which defines the geometry of the finished network.
7. The device was plasma-etched and then wet-etched to release it from the wafer, producing the completed SSN.

**Figure 12:**
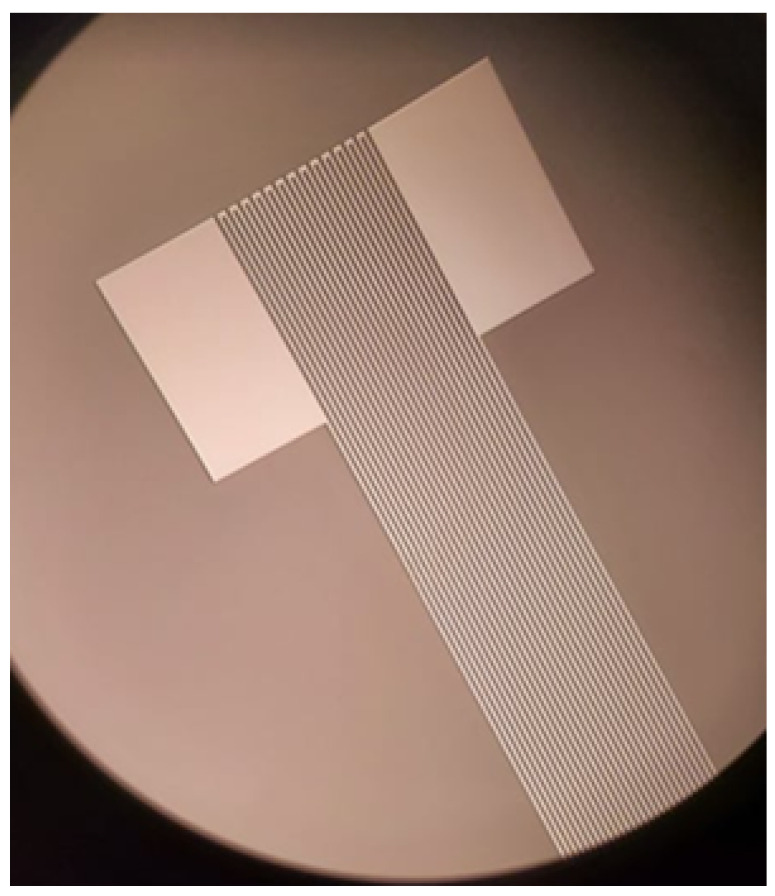
An RTD geometry was patterned to a photoresist.

The two sensor types tested in this study include RTDs and PZTs. RTDs were fabricated at the Stanford Nanofabrication Facility and comprise a platinum-resistive geometry connected to the neighboring interconnects by gold pads. This geometry is shown as patterned to a photoresist in Figure 12.

**Figure 13:**
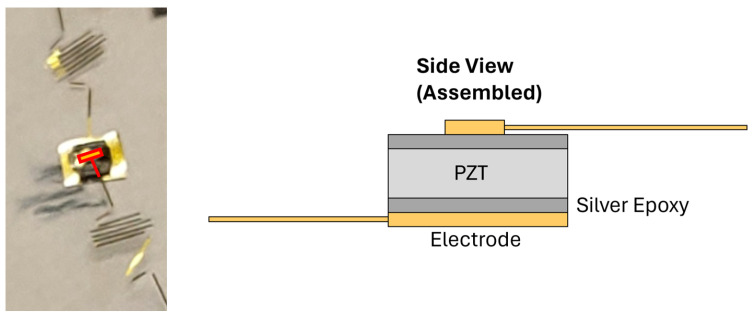
A PZT attached to gold pads (**left**). Side-view figure of PZT assembly (**right**).

For PZTs, gold pads were patterned in the SNF and then manually attached to individual PZT units. This configuration is shown in Figure 13.

### 5.2. Polar Stretch Tool

During the deployment process, the tool must manipulate the nodes at the border of the network, sweeping each of them through a path that completes the deployment process. There are several technical hurdles that must be overcome to execute this process successfully.

In taking inspiration from the quadrilateral stretch tool in previous studies performed by SACL, a polar tool may be designed.

In the quadrilateral case, the expansion must be controlled such that the edge nodes are equidistant from one each other and always trace out a perimeter with an identical aspect ratio. In the circular case, the requirements are somewhat similar in the following ways:

1. The edge nodes are radially symmetric and must remain equidistant from one another.
2. The edge nodes will always lie on a circle with the same center (i.e., will always trace out similar geometries).

**Figure 14:**
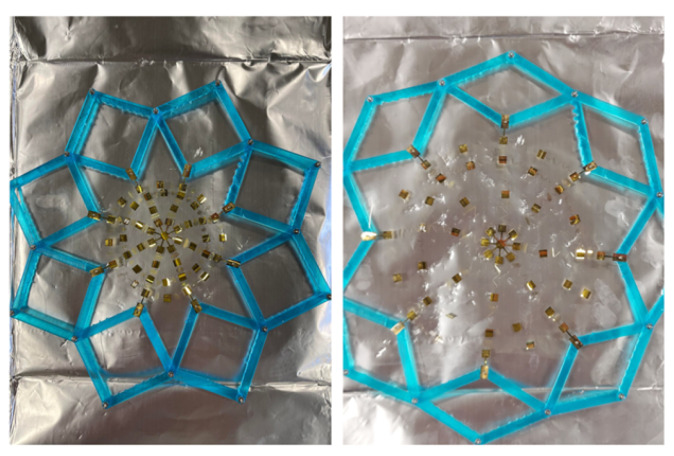
The network is expanded using the expanding ring device.

The scissor mechanism strategy that was implemented in the quadrilateral case is still attractive but needs to be modified to fit the new polar requirements. To satisfy this condition, the expanding ring device shown in Figure 14 was introduced.

The interior hinges of the device are of special interest. These hinges telescope along a circular perimeter that has an adjustable length depending on the configuration of the tool. In the case of the polar network expansion, the device is contracted to the design footprint of the tool and then expanded to the “stretched” footprint of the tool in preparation for the deployment. Figure 14 demonstrates this process.

The device has the added benefit of assisting in the wrapping of the network around the object in addition to the expansion of the network. At this stage, it is useful to note that the deployment tool and process were developed in tandem with the simulation tool; knowledge of the deployment path produced by this tool is necessary to inform the boundary conditions of this simulation. During deployment, the following process takes place:

1. The network is expanded from its design footprint to its deployment footprint.
2. The network is positioned such that the center node comes into contact with the pole of the hemispherical portion of the prepared form.
3. The tool is swept straight downward until it is in line with the base of the form.
4. The tool is contracted to bring the network in contact with the sidewall.

With the tool and deployment process designed, attention must be brought to the embedding process, which has process steps that take place before and after deployment.

### 5.3. Embedding the SSN in a Compliant Skin

As per the skin analogy given at the beginning of this work, the “nervous system” analogue has been designed and manufactured, and a process for deploying it on an object has been developed. What remains is to realize the skin material portion of the design. The previous major tasks comprise the design, manufacture, and deployment of the sensor network; at this stage in the manufacturing process, the remaining tasks are to embed the SSN in a compliant skin and then integrate it with a data acquisition device. This is accomplished with care to ensure that the skin is fabricable on a non-developable geometry.

In developing the coating and embedding process, some known methods used in the industry to coat objects with arbitrary surface geometries were examined. In general, these can be divided into three broad categories: brush coating, spray coating, and dip coating.

In brush coating, the surface of a form is brushed with the coating, which is allowed to set on the form’s surface. This method of coating is of special interest in the special effects community, where liquid latex is brushed in layers on the surface of forms or even on human skin to produce masks, prosthetics, and skin replicas. Skins fabricated with this method can provide a sufficiently thick geometry to encapsulate the sensors in this study. Brush coating is also used to conformally coat electronics such as circuit boards in order to protect, mask, or weatherproof them; while compatible with the skin fabrication process, this method typically only provides a very thin conformal layer.

Likewise, spray coating techniques use a sprayed-on coating to cover arbitrary geometries. Like with brush coating, conformal coatings for electronics can be spray-coated and typically provide a layer that is too thin for consideration in the skin fabrication process.

Finally, dip coating entails the dipping of the entire geometry into a fluid, which then sets on the form to form a skin. Dip coating is used to conformally coat electronics with thin layers of material, but can also be performed with more viscous coatings, which allows for a skin of appropriate thickness to be deposited on the target object.

In order to determine the appropriate coating, experiments with “dummy” SSNs laser cut from plastic were performed. Each experiment entailed the coating of a dummy form with the material, and an attempted embedding of the dummy SSN within that material on the surface of the form. The relative merits of each coating, as well as the coating that was elected for the final manufacturing process, are discussed in the next section.

### 5.4. Survey of Materials

The following candidate materials were tested in search of an appropriate skin substrate. The general approach during experimentation was to first coat a form in a thin layer of the material, then wrap the network around the form, and then apply a top coat of material to complete the embedded SSN skin. The following were considered for this process:

- Super-X (brush-applied adhesive);
- Silicone (two-part mold-making silicone system);
- Brush-applied silicone (one part silicone system);
- Aerosol adhesives;
- Latex rubber (dipping-applied coagulant latex system).

To summarize, brush-applied systems were found to be unsuitable for the skin fabrication process, as the brush application process tended to damage or otherwise disturb the network during application. The mold-making silicone was inappropriate for the application due to its poor thickness control, and the aerosol adhesives did not cure to a thick enough layer to form a skin. The dipping-applied latex system was selected for fabrication. Latex materials of this type are available in a variety of formulations and are used to produce latex products such as gloves, condoms, and balloons. This material shows a number of advantages: the thickness of the latex skin can be controlled depending on the formulation and certain process parameters, the latex skin does form the required tacky adhesion layer, and the latex is applied by dipping, causing minimal disturbance to the SSN after it is wrapped around the bottom coat.

### 5.5. Coating Process

In this section, dip coating and its relationship with the smart skin manufacturing process are elaborated on. Latex glove manufacturing serves as a useful example for understanding this process, which proceeds as follows:

1. A ceramic form is prepared for the dip process.
2. The form is dipped in a liquid coagulant, allowing the latex to gel on the surface of the form in the next step.
3. The coagulant-coated form is dipped into a vat of latex, which sets in combination with the coagulant on the surface of the form to a gel-like consistency. The form is allowed to reside in the latex vat for some time, depending on the desired latex thickness.
4. The latex-coated form is “leached” by dipping it into a hot water tank, where it resides until all undesirable proteins are removed from the latex skin.
5. The leached latex skin is dried in an oven below the curing temperature of the latex to remove excess moisture.
6. The dried latex skin is cured in an oven, forming the desired latex article.
7. The article is inverted from the form and is ready for use.

Usefully, steps 2–4 can be repeated, depositing successive layers of latex on a single article. In the industry, this is typically performed when the desired latex thickness is greater than achievable with a single dip. However, in the case of smart skin manufacturing, it also provides an opportunity to embed the net-like SSN between two layers of latex, which can then be co-cured to form a single skin with an enclosed SSN.

This latex dip process was modified slightly for smart skin manufacturing in the following ways:

1. Rather than a ceramic form, a 3-D printed form is used.
2. The leaching step is removed so as to minimize the risk of damage to the embedded electronics.
3. The curing schedule is performed at a lower temperature for a longer time to prevent the conductive epoxy in the PZT devices from flowing.
4. Two successive coats are used to enclose the SSN, heretofore referred to as the “base” (first) and “top” (second) coats.

This modified process is summarized in the following sections.

#### 5.5.1. Base Coat Dip

First, the 3-D printed form must be dipped in a latex coagulant. In this case, a coagulant and latex formulation were selected in consultation with a supplier (Killian Latex, Akron, OH, USA). Together, this system forms a latex skin with nominal thickness of 1 mm per dip (2 mm for the complete skin). The 3-D printed form is first manually dipped in a mixing cup filled with coagulant and allowed to reside in the coagulant for 30 s, and then, after being withdrawn from the cup, the form is gently rotated to ensure complete, uniform coverage.

**Figure 15:**
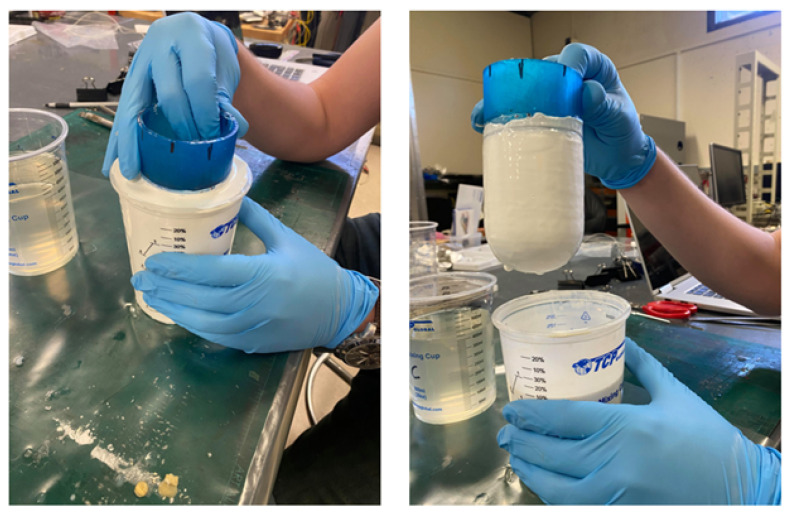
A latex coating is applied to the form by dipping.

The covered form is then dipped in a second mixing cup, this time filled with the fluid latex; care was taken to slowly insert and withdraw the form into and from this mixture to avoid thickness control issues. After experimentation, a residence time of 7 min was found to produce the desired 1 mm thickness. Figure 15 shows the latex dipping process with a 3-D printed form.

After dipping, the latex is allowed to set for approximately one minute, at which point it sets to a gel-like consistency. Critically, this gel layer has a tacky finish, which allows the sensors and interconnects to stick to the form as per the physical simulation described in this work; nodes are lightweight enough to remain attached to this coating without sticking. In some cases, small burrs appear on the surface of the skin due to dripping coagulant, which can be removed with snips after the coat has gelled.

#### 5.5.2. Sensor Network Deployment

**Figure 16:**
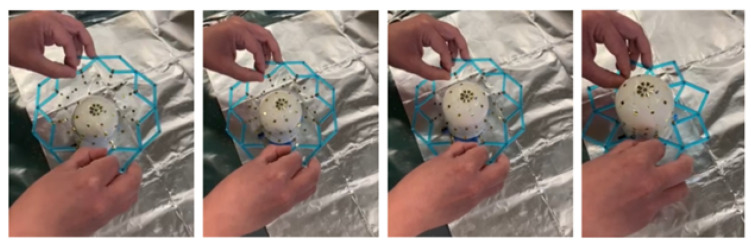
The SSN is deployed on the form.

After the base coat is in place, the network is deployed using the expanding ring-like device, which is guided around the form geometry in an open configuration, and then closed around the base of the form. Tweezers are then used to detach the edge nodes from the device and place them on the base coat. Figure 16 shows a time lapse of this process.

#### 5.5.3. Topcoat Dip

**Figure 17:**
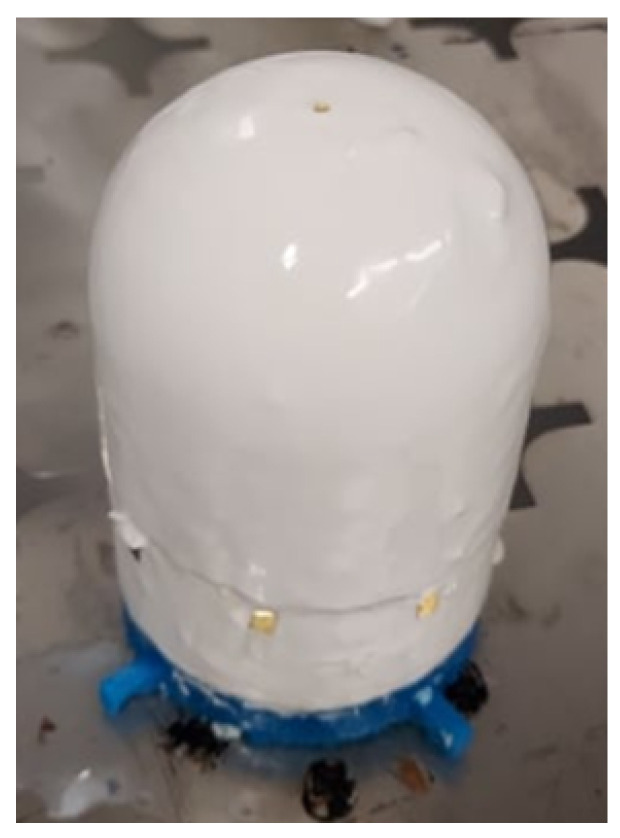
The topcoat is applied such that the edge nodes are exposed.

The topcoat is applied much like the base coat, but with one exception. To ensure that the skin can be connected to a data acquisition device, the top coat does not cover the edge pads of the SSN. Figure 17 shows a network after topcoat application with edge nodes exposed.

#### 5.5.4. Curing

**Figure 18:**
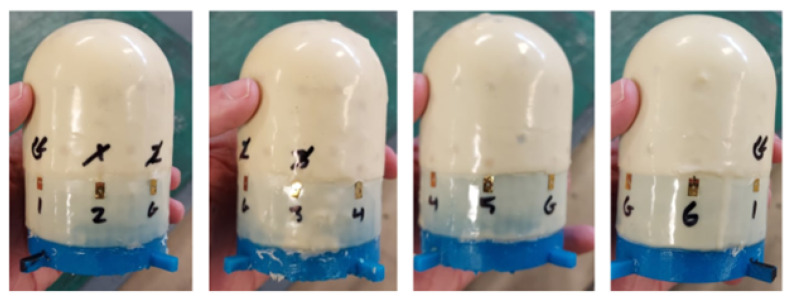
The completed skin, as viewed from each side of the form, is shown.

The latex skin is then cured in a convection oven at 70 °C for 2 h. At this temperature, the silver paste used to attach PZTs to the network does not flow. The completed smart skin is then removed from the oven. Figure 18 shows an example skin before integration with a data acquisition device.

At this stage, the device is ready for integration and validation.

### 5.6. Integration

The device, as prepared for the integration phase, comprises a deployed sensor network encapsulated within a latex skin. During the dipping process, care was taken to expose the eight outermost nodes of the network, or “edge pads”, which rest on the base coat of the latex but are not covered by the topcoat. These nodes were connected to the data acquisition device during the integration process. In each network deployed during this study, two pads were left as common grounds for multiple sensors, while the remaining pads corresponded to the signals from a single sensor.

To integrate the sensor network with a data acquisition unit, it was desired that each signal and ground path be routed from the skin through a ribbon cable; this cable could then be plugged into a data acquisition unit for processing. The integration process was as follows:

1. A piece of copper tape was placed on the base coat of the latex underneath each signal pad. Each piece of copper tape would accommodated attachments to the edge pad and ribbon cable wire.
2. Each edge pad was attached to its respective copper tape piece through painting using silver paste.
3. One side of the ribbon cable wire was stripped. These wires were cut to shape to accommodate the curvature of the form.
4. Each ribbon cable wire end was attached to its respective copper tape piece via soldering.

**Figure 19:**
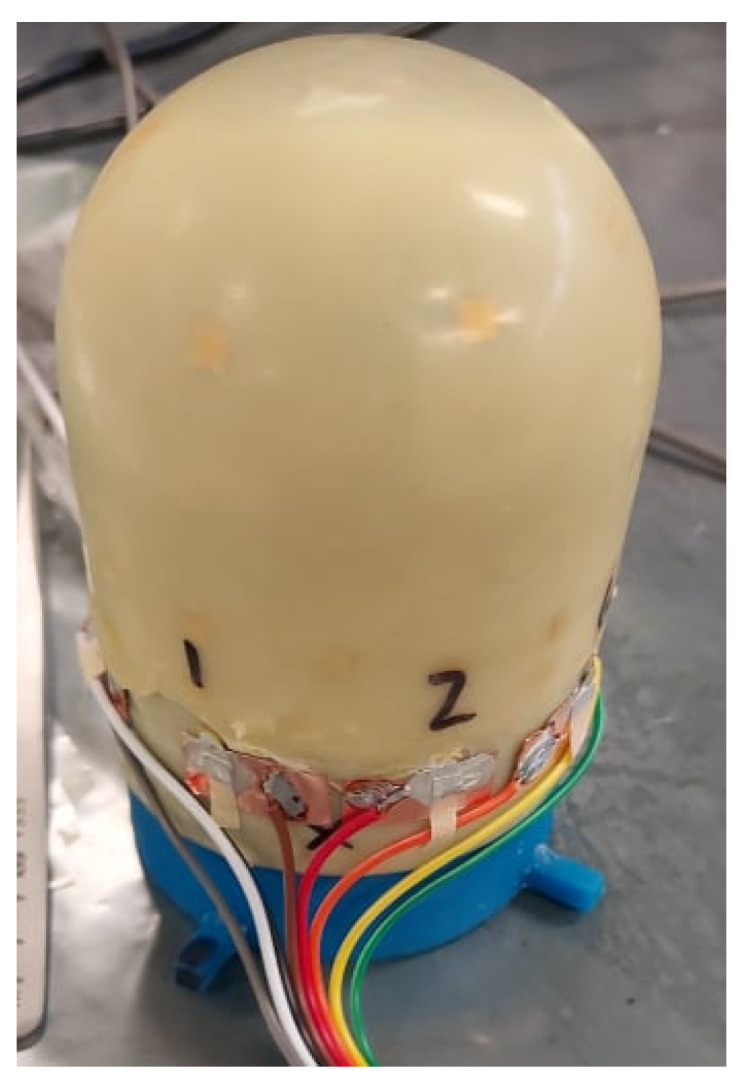
The skin was integrated with a ribbon cable.

After this process was performed, the circuit from the sensor to the ribbon cable connected the sensor to the copper tape piece through a silver paste connection. It then continued out the other end of the ribbon cable through the soldered joint as in Figure 19.

At this stage, the ribbon cable can be simply plugged into the data acquisition unit.

## 6. Results

### 6.1. RTD Validation

**Figure 20:**
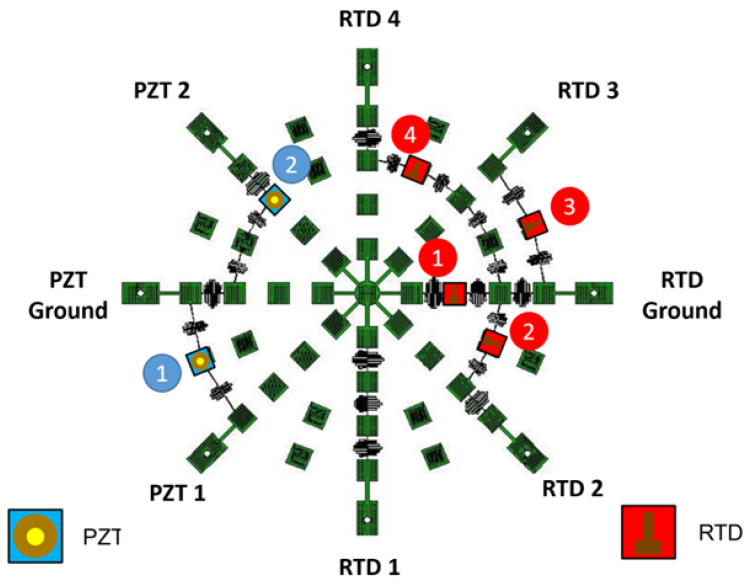
The network under test was designed with 2 PZTs (blue) and 4 RTDs (red).

To validate the performance of RTDs in the smart skin environment, a skin was manufactured with 4 RTDs on one network with signal paths shown in Figure 20.

The expected behavior of the RTDs is a linear relationship between the resistance and temperature of each RTD throughout the operating regime of each RTD sensor. To validate this behavior, a simple experiment was performed to construct a calibration curve for each RTD.

- The smart skin assembly was placed in a large oven to allow for a temperature-controlled environment. The assembly was plugged into a data acquisition unit through a voltage divider circuit by wiring it through an orifice in the rear of the oven.
- The oven was set to a prescribed set point.
- The resistance across each RTD element was calculated from the voltage divider circuit using a LabVIEW GUI and recorded.

**Figure 21:**
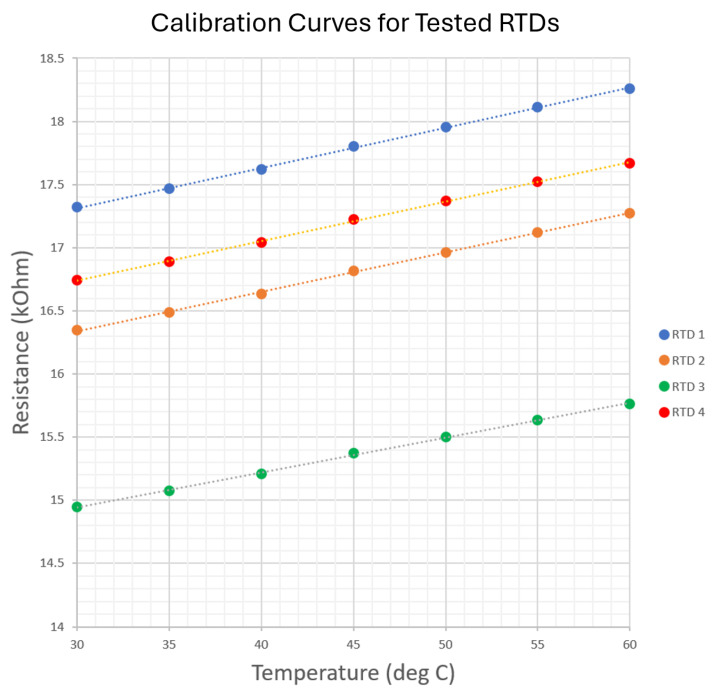
All four RTDs with linear calibration curves.

This process was performed for a series of set points to construct calibration curves for each RTD over the operating range of the sensor. The RTDs used in this study used a platinum layer patterned as previously shown in Figure 12, which acted as a thermally sensitive resistive element. In the temperature regime tested in this study, the functioning RTDs were expected to have a linear temperature sensitivity over the entire temperature range. Figure 21 shows a chart with the calibration curves for the four RTDs.

The resulting data show a linear calibration curve for each sensor as expected, and are all linear fits with $R^{2}>0.999$.

### 6.2. PZT Validation

In order to validate the PZT performance, stimuli were introduced to the smart skin with the PZT traces connected to an oscilloscope. Stimuli presented included touching with a fingertip, striking with an impact hammer, and wiring a signal through one PZT to be detected by a second PZT. While ome PZT functionality was observed in the smart skin, signals were detectable during touch tests, but the data obtained from these tests were not able to be quantified to localize the position of each touch as desired.

Several hypotheses were put forward to explain this behavior, and each was tested. The PZT mounting process shown in Figure 13 has been manually performed at the time of this writing, presenting the possibility that an incomplete circuit could exist through a mounted PZT-silver paste assembly. A number of silver epoxies were tested. The yield rate of the mounting process was approximately 50%. In successful cases, reading the PZT with a multimeter indicates its nominal capacitance. In the experimented-on skin, this nominal capacitance was observed with both PZTs. It was thus unlikely that the PZT was responding as observed during testing due to an incomplete circuit.

Secondly, it was posited that the coagulant present during the dipping step was attacking the silver paste interface. To validate this hypothesis, a network was manufactured with PZTs coated in an aerospace epoxy. Coating the PZTs in epoxy resulted in a watertight seal. Yet, the PZT response remained the same despite this coating technique.

Further study is required to refine the method used to mount the PZTs to the target object. It is possible that the limited functionality of the PZTs is likely due to the interface between the latex skin and the PZT units. In previous successful sensor deployments in experiments at SACL, the PZT was connected to the substrate with a strong epoxy bond, whereas in this case, the PZT was simply embedded between two layers of latex rubber. A latex system with greater hardness should be investigated for use in future smart skins.

## 7. Conclusions

Over the course of this work, several efforts were undertaken, resulting in the manufacture of a functional smart skin capable of detecting touch and temperature deployed over a demonstrator form. In particular, the following were performed:

1. A physics-based particle swarm optimization method was used to design the SSN, selecting the correct wire stiffness values for accurate deployment.
2. A specialized tool was developed to expand and deploy the network from its fabricated footprint onto the target object.
3. Materials were surveyed for use in the skin, and a dip-coating method was designed and implemented to form the skin layer.
4. The skin was integrated with a data acquisition unit, and sensor functionality was validated.

### Future Work

While the particle swarm optimization used in this study was successful for designing a radially symmetric network starting from a proposed configuration, future studies should be aimed at generalizing it for more complex network configurations that include asymmetry. Likewise, a tool should be developed with the purpose of generating proposed initial configurations to more generally conformally cover surfaces.

In order to improve the functionality of the PZTs in the completed skin, efforts should be undertaken to automate the PZT mounting process (e.g., with screen printing) and integrate it with the rest of the fabrication process for better process control. Additionally, dippable rubber substrates with greater hardness should be identified for future experimentation and use with the smart skin platform developed in this study. Novel epoxies and compliant substrates with sufficient hardness for this task should be surveyed.

## Data Availability

Data are contained within the article.

## Abbreviations

The following abbreviations are used in this manuscript:

SACL	Structures and Composites Lab;
SNF	Stanford Nanofabrication Facility;
RTD	Resistance Temperature Detector;
PZT	Lead Zirconate Titanate (Transducer);
PSO	Particle Swarm Optimization.
